# Comparative Effectiveness of Reperfusion Strategies in Patients with ST-Segment Elevation Myocardial Infarction: A Secondary Analysis of the Acute Coronary Syndrome Quality Improvement in Kerala (ACS QUIK) Trial

**DOI:** 10.5334/gh.868

**Published:** 2020-10-12

**Authors:** Haitham Khraishah, Barrak Alahmad, Eric Secemsky, Michael N. Young, Ahmed ElGuindy, Mark J. Siedner, Mohamad Kassab, Dhaval Kholte, Khuzeima Khanbhai, Mohamed Janabi, Kevin Kennedy, Mazen S. Albaghdadi

**Affiliations:** 1 Department of Medicine, Beth Israel Deaconess Medical Center, Harvard Medical School, Boston, MA, US; 2 Cardiovascular Research Center, Division of Cardiology, Massachusetts General Hospital, Harvard Medical School, Boston, MA, US; 3 Environmental Health Department, Harvard T.H. Chan School of Public Health, Harvard University, Boston, MA, US; 4 Richard A. and Susan F. Smith Center for Outcomes Research in Cardiology, Beth Israel Deaconess Medical Center, Boston, MA, US; 5 Cardiology Division, Dartmouth-Hitchcock Medical Center, Geisel School of Medicine, Dartmouth, Lebanon, NH, US; 6 Department of Cardiology, Aswan Heart Centre, EG; 7 Division of Infectious Diseases and Medical Practice Evaluation Center, Massachusetts General Hospital, Harvard Medical School, Boston, MA, US; 8 Department of Adult Cardiology, Jakaya Kikwete Cardiac Institute, Dar es Salaam, TZ; 9 Mid America Heart Institute, St Luke’s Hospital, Kansas City, Missouri, US

**Keywords:** ST-segment elevation myocardial infarction (STEMI), reperfusion strategies, low- and middle-income countries (LMICs), comparative effectiveness

## Abstract

**Introduction:**

Substantial heterogeneity exists in reperfusion strategies for patients with ST-segment myocardial infarction (STEMI) in low- and middle-income countries (LMICs). We sought to compare outcomes associated with primary percutaneous coronary intervention (PPCI) and non-primary percutaneous coronary intervention (nPPCI) reperfusion strategies in patients with STEMI in Kerala, India.

**Methods:**

We performed a retrospective analysis of patients with STEMI (n = 8665) from the Acute Coronary Syndrome Quality Improvement in Kerala (ACS QUIK) randomized trial receiving either PPCI (n = 6623) or nPPCI (n = 2042). nPPCI included all PCI strategies implemented when PPCI was not available including all post-fibrinolysis PCI strategies and PCI without fibrinolysis. Clinical outcomes among patients undergoing PPCI and nPPCI were compared after propensity-score matching. The main outcomes were the rates of in-hospital and 30-day major adverse cardiovascular events (MACE), defined as the composite of death, reinfarction, stroke, and major bleeding.

**Results:**

In the propensity-score matched cohort (n = 1266 in each group), nPPCI had longer symptom onset to hospital arrival time (347.5 vs. 195.0 minutes, p < 0.001), door to balloon time (108 minutes vs. 75 minutes, p < 0.001), and were less likely to receive a coronary stent (89.4% vs. 95%, p < 0.001), including drug-eluting stents (89.5% vs. 94.4%, p < 0.001). There were no clinically meaningful differences in discharge medical therapy. However, patients treated with nPPCI were less commonly referred for cardiac rehabilitation (20.2% vs. 24.2%; p = 0.019). In-hospital (3.6% vs. 3.3%, p = 0.74%) and 30-day (4.4% vs. 4.6%, p = 0.77) MACE did not differ between nPPCI and PPCI matched groups.

**Conclusion:**

In a large, contemporary population of STEMI patients from a LMIC, patients treated with a nPPCI reperfusion strategy had comparable short- and intermediate-term outcomes compared to PPCI despite differences in hospital presentation time and coronary stent use. These findings are reassuring but highlight the need for continued quality improvement in the delivery of STEMI care in resource-limited settings.

## Introduction

Atherosclerotic cardiovascular disease (CVD) remains the leading cause of death worldwide with 80% of cardiovascular deaths occurring in lower- and middle- income countries (LMICs) [1,2,3]. ST-segment elevation myocardial infarction (STEMI) represents nearly 60% of all acute coronary syndrome (ACS) cases in South Asia [1,4,5]. Primary percutaneous coronary intervention (PPCI) remains the recommended reperfusion strategy in patients with STEMI [6,7]. However, the efficacy of PPCI is limited in patients not receiving PCI within 120 minutes of first medical contact [7]. In LMICs, timely PPCI is difficult to achieve due to a paucity of PCI-capable hospitals, delayed recognition of ischemic symptoms, limited means of safe and timely transfer, cost considerations, and a host of other system-level barriers [8].

Pharmacoinvasive PCI (PhI) is an alternative reperfusion strategy that involves the administration of fibrinolysis followed PCI usually within 24 hours [9]. When PPCI cannot be achieved in a timely manner, PhI has been shown to result in similar ischemic and bleeding outcomes compared to PPCI [10]. Although STEMI systems of care in LMICs are evolving due to the implementation of quality improvement programs that improve access to PCI [11], the management of acute coronary syndromes (ACS) in resource-limited settings is heterogeneous and must frequently adapt to system- and patient-level factors that may hamper strict adherence to guideline-recommended care [4]. Furthermore, studies demonstrating equivalence between PPCI and PhI were primarily conducted in high-income countries (HIC) with well-established systems of care for managing STEMI [12]. Patient- and system-level factors may limit the ability of emerging healthcare systems to deliver timely fibrinolysis, PhI, or PPCI for STEMI patients in LMICs, and therefore patients may receive reperfusion strategies that reflect medical and logistical considerations unique to the patient and healthcare setting. Thus, contemporary data are needed to provide insights into gaps in guideline-based STEMI management and identify opportunities for quality improvement in LMICs. Hence, the aim of this study is to compare clinical outcomes of patients with STEMI undergoing guideline-based PPCI compared to patients receiving non-primary PCI (nPPCI) reperfusion in a resource-limited setting among patients from the Acute Coronary Syndrome Quality Improvement in Kerala (ACS QUIK) randomized trial.

## Methods

### Study population

**Figure 1:**
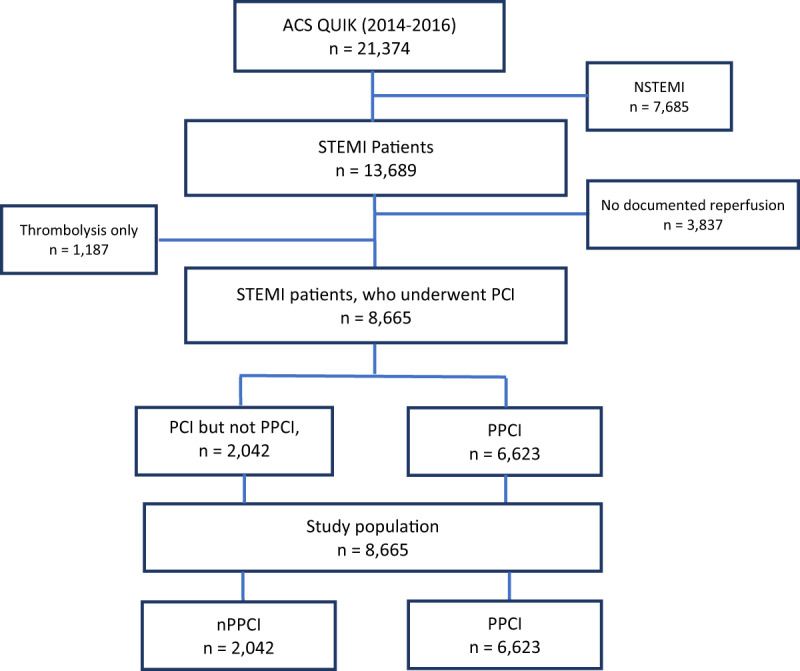
The study flow diagram of patients. ACS QUIK indicates Acute Coronary Syndrome Quality Improvement in Kerala; STEMI, ST-segment myocardial infarction; NSTEMI, non-ST-segment myocardial infarction; PCI, percutaneous coronary intervention; PPCI, primary percutaneous coronary intervention; nPPCI, non-primary percutaneous coronary intervention.

We obtained the ACS QUIK study data from the Biologic Specimen and Data Repository Information Coordinating Center (BioLINCC; National Heart, Lung, and Blood Institute, Bethesda, Maryland). The design and primary results of the trial have been previously published [13,14]. In brief, 63 hospitals in Kerala, India participated in a cluster randomized, stepped-wedge clinical trial to evaluate the impact of a locally adapted quality improvement toolkit to improve ACS outcomes [15]. The trial included 21,374 ACS patients between November 10, 2014 and November 9, 2016. The ACS QUIK trial received ethics board approval from local, national, and international bodies and was approved by the Indian Health Ministry Screening Committee [13,14] and this sub-study was approved the Partners Healthcare Institutional Review Board. For this secondary analysis, we focused on STEMI (n = 13,689) patients who received any type of reperfusion strategy (n = 9,852) and analyzed those patients who underwent PCI during their hospitalization (n = 8665) including patients who underwent nPPCI (n = 2042) or PPCI (n = 6623) as defined below (Figure 1).

### Reperfusion Strategy Definitions

In the ACS QUIK trial, systematic adjudication was performed to confirm that patients received PPCI and PCI, including patients transferred to PCI-capable hospitals following fibrinolysis. PPCI was defined as PCI within 90 minutes from first medical contact (FMC) to arrival to a PCI capable facilities or 120 minutes from FMC if interhospital transfer was involved. Door-to-balloon time was defined as the time from arrival to a PCI-capable facility to balloon inflation. However, the administration of fibrinolysis followed by PCI at the same hospital and door-to-needle time were not systematically adjudicated, and thus details on the specific type of post-fibrinolytic PCI strategy were not available, including rescue PCI (i.e., emergent PCI for failed fibrinolysis), pharmacoinvasive PCI (i.e., angiography with possible PCI within four to 24 hours following fibrinolysis), and facilitated PCI (fibrinolysis delivered prior to a planned PCI). Furthermore, inconsistencies (data not shown) were observed between hospitals in the documentation of reperfusion strategies including the documentation of rescue PCI and PPCI without prior administration of fibrinolysis for a given patient. Accordingly, nPPCI was defined as all PCI strategies implemented when PPCI was not available including the performance of all post-fibrinolytic PCI strategies defined above and PCI that did not involve the prior administration of fibrinolysis and did not occur within the timeframes defined by PPCI. Thus, we grouped patients into two reperfusion groups: 1) non-primary PCI (nPPCI) and 2) primary PCI (PPCI) (Figure 1). This stratification may be more reflective of real-world reperfusion strategies implemented in LMICs and also attempts to reconcile the limitations of the data.

### Outcomes

The primary outcomes of this study were in-hospital and 30-day major adverse cardiovascular events (all-cause death, reinfarction, stroke and major bleeding). The 30-day outcomes were defined as any major event that occurred either in-hospital or anytime up to 30 days from discharge. Major bleeding was defined according to the Global Utilization of Streptokinase and Tissue Plasminogen Activator for Occluded Coronary Arteries [GUSTO] criteria [16]. Other outcomes assessed included post-STEMI left ventricular ejection fraction (EF) on echocardiography, and in-hospital incident heart failure, cardiogenic shock, and cardiac arrest.

### Statistical analysis

Continuous variables were summarized by the mean and standard deviation (SD) or median and interquartile range (IQR). For normally distributed variables, student t-test was used, while Mann-Whitney U test was used for data that was not normally distributed. Categorical variables are reported as numbers and percentages and computed using Chi-square. To account for the non-random allocation of nPPCI vs. PPCI, we used a propensity score matching strategy. The propensity score was derived using logistic regression predicting PPCI based on: cluster randomization, age, sex, transfer status, insurance, heartrate, SBP, weight, smoking status, diabetes, Killip class, the presence of on-site catheterization laboratory, hospital size, and hospital type. We then used a nearest neighbor matching algorithm with a caliper width of 0.2 times the standard deviation of the logit of the propensity score [17]. The success of the match was checked using standardized differences with values <10% indicating balance. Outcomes were then compared in the unmatched and matched cohorts using relative risk (RR). Finally, we tested various subgroups of interest, including age, gender, Killip class, hospital size, symptoms onset time, culprit vessel, hypertension, diabetes, and smoking. A logistic regression model was performed to test the interaction between treatment modality and subgroup of interest. We then reported out the effect of treatment modality within subgroup using odds ratios and 95% confidence intervals. The alpha level for statistical significance was set at 0.05 and all analyses were done with SAS 9.4 (Cary, NC).

## Results

### Baseline Characteristics of Patients and Hospitals by Reperfusion Group

A total of 8665 STEMI patients from the parent study were eligible for this analysis, 2042 of which underwent nPPCI (Figure 1). Patients who underwent nPPCI were more likely to be men, present later after the onset of chest pain (median of 300 vs. 159 minutes for PPCI; p < 0.001), and have lower Killip class and troponin on presentation (Table 1). System level differences between the PPCI and nPPCI groups were apparent with respect to the hospital type, size, and the availability of a cardiac catheterization laboratory between patients treated with PPCI and nPPCI (Table 2). In the unmatched group, nPPCI patients were more likely to present to smaller hospitals (≤ 200 beds) (12.6% vs. 4.1%; p < 0.001), extra-large hospitals (>1000 beds) (33.7% vs. 21.7%; p < 0.001), non-PCI capable hospitals (6.7% vs. 0.8%; p < 0.001) and governmental hospitals (37.7% vs. 28.9%; p < 0.001). Following propensity-score matching (n = 1266 in each arm), baseline characteristics were similar with standardized differences <10%, except for the time from symptom onset to presentation, which was longer in the nPPCI group (347.5 min vs. 194 min; p < 0.001).

**Table 1:** Baseline Characteristics of ACS QUIK STEMI Patients Stratified by Reperfusion Strategy.

	All Patients		p-value	Propensity-Matched Patients		p-value
	
	Non PPCI (n = 2042)	PPCI (n = 6623)		Non PPCI (n = 1266)	Primary PCI (n = 1266)	

Age (years), mean (SD)	57.3 (11.0)	58.1 (11.3)	0.004	57.1 (11.2)	57.2 (11.5)	0.796
Female sex, n (%)	338 (16.6)	1291 (19.5)	0.002	219 (17.3)	230 (18.2)	0.567
Weight (kg), mean (SD)	66.1 (9.3)	64.7 (9.5)	<0.001	65.4 (10.3)	65.1 (9.8)	0.442
Heart Rate (beats/min), mean (SD)	77.5 (17.6)	77.1 (16.8)	0.248	78.2 (17.0)	78.1 (18.0)	0.891
Systolic BP (mmHg), mean (SD)	135.1 (26.6)	136.1 (27.4)	0.167	136.2 (27.4)	135.4 (27.5)	0.454
No insurance, n (%)	1168 (57.2)	4351 (65.7)	<0.001	959 (75.8)	945 (74.6)	0.519
Initial troponin (ng/mL), median [IQR]	1.4 (0.2, 7.7) (n = 570)	2.4 (0.3, 10.0) (n = 1785)	0.054	2.0 (0.3, 9.4) (n = 486)	1.9 (0.3, 8.7) (n = 439)	0.135
LDL (mg/dL), mean (SD)	121.0 (38.6) (n = 1616)	127.1 (41.0) (n = 4668)	<0.001	122.3 (42.1) (n = 997)	127.8 (41.4) (n = 974)	0.003
Triglycerides (mg/dL), mean (SD)	128.7 (71.9) (n = 1619)	136.0 (73.7) (n = 4675)	<0.001	134.1 (72.5) (n = 1001)	130.8 (69.2) (n = 974)	0.300
Fasting glucose (mg/dL), mean (SD)	157.8 (69.2) (n = 1430)	151.5 (65.6) (n = 4049)	0.002	155.0 (68.0) (n = 899)	157.2 (69.0) (n = 864)	0.506
Creatinine (mg/dl), mean (SD)	1.1 (0.4) (n = 1309)	1.0 (0.4) (n = 4489)	<0.001	1.1 (0.5) (n = 792)	1.1 (0.4) (n = 747)	0.227
Hemoglobin (g/dl), mean (SD)	13.8 (1.9) (n = 2025)	13.6 (1.8) (n = 6460)	<0.001	13.6 (1.8) (n = 1250)	13.7 (1.9) (n = 1235)	0.325
Killip Class, n (%)						
1	1862 (91.2)	5915 (89.3)	0.005	1117 (88.2)	1150 (90.8)	0.200
II–IV	180 (8.8)	708 (10.7)		149 (11.8)	116 (9.2))	
Time from symptom onset to hospital arrival (min), median [IQR]	300.0 (135.0, 870.0) (n = 1961)	159.0 (100.0, 365.0) (n = 6406)	<0.001	347.5 (130.0, 1020.0) (n = 1200)	195.0 (105.0, 510.0) (n = 1226)	<0.001
Risk factors, n (%)						
Smoking, n (%)	693 (33.9)	2145 (32.4)	0.191	374 (29.5)	396 (31.3)	0.341
Hypertension, n (%)	821 (40.2)	2580 (39.0)	0.311	529 (41.8)	504 (39.8)	0.312
Diabetes mellitus, n (%)	869 (42.6)	2652 (40.0)	0.043	576 (45.5)	553 (43.7)	0.357
History of cerebrovascular accident, n (%)	22 (1.1)	84 (1.3)	0.49	17 (1.3)	16 (1.3)	0.860

LDL, low-density lipoprotein cholesterol. IQR, interquartile range.

**Table 2:** Hospital of Presentation Characteristics.

	All Patients		p-value	Propensity-Matched Patients		p-value
	
	Non PPCI (n = 2042)	PPCI (n = 6623)		Non PPCI (n = 1266)	PPCI (n = 1266)	

Hospital Characteristics						
Hospital type, n (%)						
Government	770 (37.7)	1911 (28.9)	<0.001	184 (14.5)	212 (16.7)	0.305
Non-profit/Charity	365 (17.9)	1993 (30.1)		296 (23.4)	285 (22.5)	
Private	907 (44.4)	2719 (41.1)		786 (62.1)	769 (60.7)	
Catheterization laboratory, n (%)						
Installed During Study (n = 3)	76 (3.7)	106 (1.6)	<0.001	46 (3.6)	36 (2.8)	0.472
No (n = 17)	136 (6.7)	55 (0.8)		28 (2.2)	32 (2.5)	
Yes (n = 43)	1830 (89.6)	6462 (97.6)		1192 (94.2)	1198 (94.6)	
Hospital size, n (%)						
Extra large (>1000) (n = 5)	689 (33.7)	1439 (21.7)	<0.001	103 (8.1)	108 (8.5)	0.672
Large (501–1000) (n = 15)	658 (32.2)	3333 (50.3)		655 (51.7)	642 (50.7)	
Medium (201–500) (n = 24)	437 (21.4)	1580 (23.9)		387 (30.6)	408 (32.2)	
Small (≤200) (n = 19)	258 (12.6)	271 (4.1)		121 (9.6)	108 (8.5)	

### Hospital and Discharge Management

In all patients, nPPCI patients were less likely to receive in-hospital aspirin (97.9% vs. 99.2%; p < 0.001), a second antiplatelet agent (98.5% vs. 99.7%; p < 0.001), and β-blockers (30.6% vs. 40.8%; p < 0.001) (Table 3). Compared to PPCI, nPPCI group were more likely to be transferred from another facility (58.7% vs. 40.1%; p < 0.001), had longer door-to-balloon time once at PCI-capable facility (medians 90 vs. 70 minutes; p < 0.001), and less likely to have stents placed (90.8% vs. 95.0%; p < 0.001). At discharge, the use of β-blocker, statins, ACEi or ARB, or referral to cardiac rehabilitation was less common among patients treated with nPPCI (Table 3).

**Table 3:** In-hospital and On-discharge Treatment Patterns Stratified by Reperfusion Strategy.

	No./Total No. (%)		p-value	No./Total No. (%)		p-value
	All Patients			Propensity-Matched Patients		
	Non PPCI (n = 2042)	PPCI (n = 6623)		Non PPCI (n = 1266)	PPCI (n = 1266)	

In-hospital Medications, n (%)						
Aspirin	1998/2041 (97.9)	6571/6621 (99.2)	<0.001	1231/1265 (97.3)	1251/1266 (98.8)	0.006
Second antiplatelet	2012/2042 (98.5)	6603/6623 (99.7)	<0.001	1241/1266 (98.0)	1261/1266 (99.6)	<0.001
Anticoagulant	1673/2036 (82.2)	5452/6615 (82.4)	0.797	947/1261 (75.1)	986/1264 (78.0)	0.084
β-blocker	615/2013 (30.6)	2658/6519 (40.8)	<0.001	519/1249 (41.6)	433/1263 (35.2)	0.001
Reperfusion details						
Transfer from another facility, n (%)	1199 (58.7)	2655 (40.1)	<0.001	616 (48.7)	632 (49.9)	0.524
Door-to-balloon time (STEMI), median [IQR], min	90 (60, 212) (N = 1493)	70 (53, 103) (n = 6168)	<0.001	108 (60, 333) (N = 802)	75 (52, 115) (n = 1165)	<0.001
Echocardiography, n (%)	1972 (96.6)	6342 (95.8)	0.102	1202 (94.9)	1202 (94.9)	1.000
Stents placed, n (%)	1854 (90.8)	6293 (95.0)	<0.001	1132 (89.4)	1203 (95)	<0.001
DES, n (%)	1696/1845 (91.5)	6092/6293 (96.8)	<0.001	1013/1132 (89.5)	1136/1203 (94.4)	<0.001
Location of stents						
LAD, n (%)	1015 (54.7)	3240 (51.5)	0.013	616 (54.4)	602 (50.0)	0.034
Discharge treatment and counseling, n (%)						
Aspirin	1981/1994 (99.3)	6412/6436 (99.6)	0.099	1225/1237 (99.0)	1227/1238 (99.1)	0.832
Second antiplatelet	1958/1994 (98.2)	6384/6442 (99.1)	<0.001	1208/1237 (97.7)	1219/1240 (98.3)	0.249
β-blocker	1140/1968 (57.9)	4537/6319 (71.8)	<0.001	812/1217 (66.7)	761/1209 (62.9)	0.051
Statin	1911/1986 (96.2)	6287/6438 (97.7)	<0.001	1169/1229 (95.1)	1201/1239 (96.9)	0.020
ACEi or ARB	696/1966 (35.4)	3664/6319 (58.0)	<0.001	521/1210 (43.1)	528/1212 (43.6)	0.801
Cardiac rehabilitation referral	251/1911 (13.1)	2120/6335 (33.5)	<0.001	232/1147 (20.2)	290/1196 (24.2)	0.019

STEMI, ST-segment myocardial infarction; DES, drug-eluting stent; LAD, left anterior descending artery; ACEi, angiotensin-converting enzyme inhibitor; ARB, angiotensin receptor blocker.

In the propensity-matched cohort, nPPCI group were less likely to receive in-hospital aspirin and a second antiplatelet therapy (Table 3); however, they were more likely to receive β-blocker (41.6% vs. 35.2% in PPCI; p < 0.001). Compared to PPCI, nPPCI patients had similar transfer rates, longer door-to-balloon time once at a PCI-capable hospital (108 vs. 75min in PPCI; p < 0.001), and were less likely to have a stent implanted (89.5% vs. 94.4% in PPCI; p < 0.001). On discharge, patients treated with nPPCI were less likely to be discharged on a statin (95.1% vs. 96.9%; p = 0.020), and the disparity in referral to cardiac rehabilitation persisted for nPPCI patients although referral rates were low overall (20.2% vs. 24.2%; p = 0.019). There were no other significant differences between the propensity matched two groups.

### Clinical Outcomes

**Figure 2:**
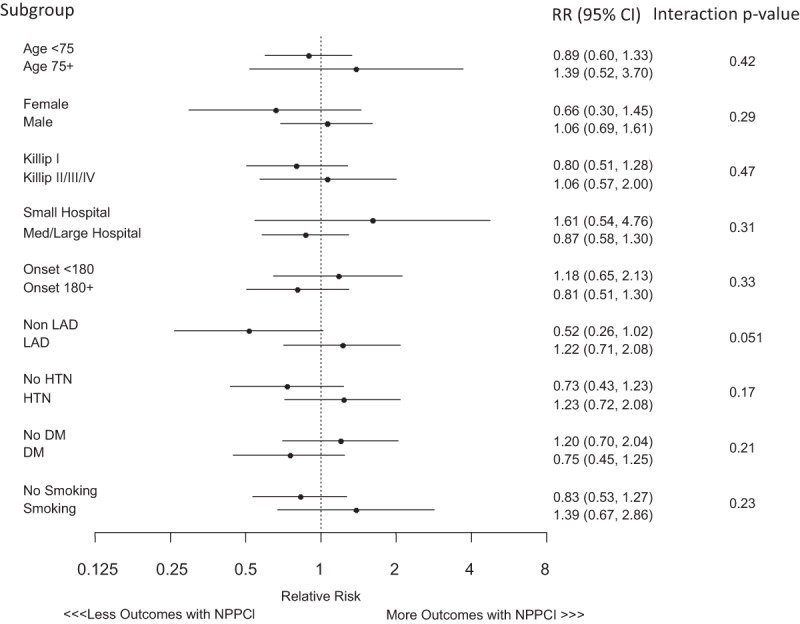
Relative Risk (RR) for in-hospital major adverse cardiac events in propensity-matched cohort according to subgroup. Onset refers to the time from symptom onset to hospital presentation. CI indicates confidence interval; LAD, left anterior descending artery; HTN, hypertension; DM, diabetes mellitus.

In-hospital and 30-day MACE were similar between nPPCI and PPCI groups in all patient and propensity-matched cohorts (Table 4). Before propensity-matching, in-hospital (1.6% vs. 2.4%, p = 0.032) and 30-day mortality (2.0 vs. 3.0, p = 0.014) were lower in the nPPCI, while the incidence of in-hospital shock (2.6% vs. 1.5%, p = 0.001) and reinfarction (1.3% vs. 0.6%, p = 0.002) were higher in in nPPCI group. In the propensity-matched cohorts, no significant difference was detected for in-hospital MACE (3.6% vs. 3.3%, p = 0.74), 30-day MACE (4.4% vs. 4.6%, p = 0.77), or any of the secondary endpoints for nPPCI compared to PPCI. In addition, we did not observe an interaction between any of the sub-groups tested and treatment with nPPCI or PPCI with respect to the incidence of in-hospital MACE with all p-values > 0.10 (Figure 2).

**Table 4:** Risk of adverse clinical outcomes among STEMI patients comparing non-PPCI to PCI.

	All Patients			p-value	Propensity-Matched Patients			p-value
	
	Non PPCI (n = 2042)	PPCI (n = 6623)	Unadjusted RR (95% CI)		Non PPCI (n = 1266)	PPCI (n = 1266)	Adjusted RR (95% CI)	

**In-hospital outcomes**								
MACE	56 (2.7)	207 (3.1)	0.88 (0.66, 1.17)	0.377	45 (3.6)	42 (3.3)	1.07 (0.71, 1.62)	0.74
Mortality	32 (1.6)	156 (2.4)	0.67 (0.46, 0.97)	0.032	24 (1.9)	23 (1.8)	1.043 (0.59, 1.84)	0.882
Shock	53 (2.6)	101 (1.5)	1.70 (1.23, 2.36)	0.001	16 (1.3)	12 (0.9)	1.333 (0.63, 2.81)	0.447
Heart failure	35 (1.7)	132 (2.0)	0.86 (0.59, 1.24)	0.422	24 (1.9)	22 (1.7)	1.091 (0.62, 1.94)	0.766
Cardiac arrest	31 (1.5)	129 (1.9)	0.78 (0.53, 1.15)	0.207	20 (1.6)	29 (2.3)	0.69 (0.39, 1.21)	0.194
Reinfarction	26 (1.3)	40 (0.6)	2.11 (1.29, 3.45)	0.002	24 (1.9)	14 (1.1)	2.43 (1.31, 4.503)	0.102
Stroke	4 (0.2)	18 (0.3)	0.72 (0.24, 2.13)	0.551	3 (0.2)	6 (0.5)	0.50 (0.13, 2.00)	0.507
Major bleeding	3 (0.1)	9 (0.1)	1.08 (0.29, 3.99)	0.99	2 (0.2)	1 (0.1)	2.0 (0.182, 22.03)	0.99
Post-STEMI LVEF (%), median [IQR]	50 (45, 60)	53 (45, 59)	1.01 (1.01, 1.02)	<0.001	52 (45, 60)	52 (45, 60)	1.00 (0.99, 1.02)	0.727
**30-day outcomes**								
30-d MACE	69/2025 (3.4)	266/6562 (4.1)	0.84 (0.65, 1.09)	0.189	55/1252 (4.4)	58/1252 (4.6)	0.948 (0.661, 1.36)	0.772
30-d mortality	41/2025 (2.0)	200/6562 (3.0)	0.67 (0.48, 0.93)	0.014	30/1252 (2.4)	32/1252 (2.6)	0.938 (0.573, 1.533)	0.797
30-d CVD mortality	40/2025 (2.0)	195/6562 (3.0)	0.67 (0.48, 0.93)	0.016	29/1252 (2.3)	31/1252 (2.5)	0.935 (0.567, 1.543)	0.793
Stroke	7/2025 (0.3)	36/6563 (0.5)	0.63 (0.28, 1.42)	0.258	5/1252 (0.4)	11/1252 (0.9)	0.455 (0.158, 1.304)	0.132
30-d major GUSTO bleeding	3/2025 (0.1)	15/6623 (0.2)	0.65 (0.19, 2.24)	0.591	2/1252 (0.2)	3/1252 (0.2)	0.667 (0.112, 3.983)	1.000
Reinfarction	29/2025 (1.4)	50/6563 (0.8)	1.88 (1.19, 2.96)	0.005	27/1252 (2.2)	18/1252 (1.4)	1.5 (0.83, 2.71)	0.175

MACE: death, reinfarction, stroke or major bleeding. Values are n (%). STEMI, ST-segment myocardial infarction; LVEF, left ventricular ejection fraction; IQR, interquartile range; CVD, cardiovascular disease; Global Utilization Of Streptokinase And Tpa For Occluded Arteries (GUSTO).

## Discussion

In the current analysis of ACS-QUIK trial, we aimed to compare the efficacy and safety of nPPCI to PPCI among patients with STEMI in a low- and middle-income country (LMIC) using a large, contemporary population of Indian patients with STEMI. We observed that despite a longer time from symptom onset to hospital arrival, door-to-balloon time, and reduced utilization of coronary stents (including drug-eluting stents) at the time of PCI, the clinical outcomes among patients treated with a nPPCI strategy (including pharmacoinvasive, facilitated and rescue PCI) were similar to PPCI in terms of the composite of death, reinfarction, stroke or major bleeding during hospitalization or at 30-day follow-up. We also did not find a significant difference between the groups in terms of incident heart failure or post-STEMI LVEF.

Our study findings on the safety and efficacy of nPPCI among STEMI patients in an LMIC are encouraging. The results from our study are similar to previously published real world experiences from HICs in North America [18,19], Korea [20], Middle East [21], and Europe [22], comparing PPCI to pharmacoinvasive PCI (PhI) strategies. The randomized STREAM trial (Strategic Reperfusion Early after Myocardial Infarction) examined 1892 patients with STEMI who could not undergo PPCI within one hour of presentation. Rates of the composite outcome of death, shock, heart failure, or reinfarction were similar at 30-days and one year among patients treated with PhI and PPCI, and bleeding rates (after a dose-reduction in fibrinolysis for patients over the age of 75 were also comparable between groups) [9,23]. The rationale behind this approach is that the initial fibrinolytic therapy would restore coronary circulation early on. Then, invasive intervention with PCI would either reopen the culprit artery in case of failed thrombolysis or augment the outcomes of a successful fibrinolysis [24]. The STREAM trial and other studies demonstrating the efficacy of PhI compared to PPCI were performed in HICs with fewer patient- and system-level barriers to STEMI care and more homogeneity of reperfusion strategies compared to LMICs. It is also important to note additional differences that have been previously reported between HICs and LMICs including that STEMI patients from India are typically younger, tend to present later after symptoms onset, and have a higher prevalence of cardiovascular risk factors [4].

India is a prototypical example of an LMIC that is going through an epidemiological transition with an increasing burden of non-communicable diseases and where CVD already accounts for more than a fourth of all deaths among individuals over the age of 25 [25]. Prior studies comparing different reperfusion strategies in patients with STEMI from India are limited by small sample size [26,27,28]. In a retrospective analysis of STEMI patients in India comparing outcomes among patients receiving PhI (n = 43) to those treated with PPCI (n = 95), Alex et al. examined 139 STEMI patients from a single hospital in South India. Similar to the findings of our study, the investigators did not find significant differences between patients treated with PhI or PPCI in the composite efficacy endpoints of death, reinfarction, and shock at 30-days or in secondary endpoints of bleeding or stroke [27].

In an encouraging study by Alexander et al. designed to address system-level factors in care delivery in Tamil Nadu, India, the investigators used a regional hub-and-spoke model to improve STEMI care through greater use of PPCI and PhI which was associated with improved mortality at 1 year [11]. Implementation of this program resulted in an increased utilization of PhI from 13% to 58% amongst patients presenting to spoke hospitals [11], and a reduction in the time to treatment after implementation (17.3 vs. 39.2 hours). In the original ACS QUIK trial, implementation of a quality improvement intervention was unfortunately not associated with an improvement in door-to-balloon time for STEMI patients [14]. In fact, door-to-balloon times were increased in the intervention compared to control group (77 vs. 65 minutes) underscoring the immense challenges of addressing the multitude of patient- and system-level factors that affect STEMI care in LMICs. In our sub-study, door-to-balloon times in patients who received nPPCI (108 minutes) were significantly shorter than those reported by Alexander et al. and may explain the lower in-hospital mortality in our study (1.9%) that those reported among spoke hospitals in the post-implementation phase in the Tamil Nadu study (6.3%) [11]. Together these results suggest that nPPCI reperfusion strategies including PhI may be a reasonable alternative for STEMI treatment in LMICs where treatment delays are common and PPCI may not be available.

Provision of high-quality STEMI care is resource-intensive and requires a well-developed infrastructure and timely coordination between hospital systems, referring providers, and emergency medical services (EMS) [29]. Orchestration of this infrastructure is challenging in a resource-limited setting due to inadequate EMS staff and training [30], geographic barriers, scarcity of trained professionals [31], and a limited number of PCI-capable facilities [32]. Thus, there is a dire need to evolve systems of care for STEMI suited to the challenges posed by these various patient- and system-level factors. Improving access to timely reperfusion is a key attribute of a successful STEMI care network. In the present study, we observed that patients treated with nPPCI presented to the hospital over five hours after symptom onset and those treated with PPCI presented for care approximately three hours after onset which is significantly greater than that observed in HIC cohorts [33]. In addition, the cost burden for STEMI care in LMICs adds another layer of complexity for fragile health care systems especially during the susceptible narrow window of time that is critical for successful reperfusion. For example, the health care system in India (at the time of the study) relied heavily on the private sector, where payments are almost entirely out-of-pocket and insurance coverage is low [34]. In our study population of Indian STEMI patients, approximately 50–60% of the unmatched cohort of patients did not have any insurance coverage and approximately 75% of the propensity matched patients did not have insurance. Furthermore, the majority of patients in our matched cohort were treated at private hospitals underscoring the challenges faced by patients with STEMI in India who are frequently lacking insurance coverage but may commonly receive care at private hospitals where government safety-net programs may be unavailable or more difficult to navigate.

Implementation of guideline-based management for ACS has improved the outcomes of STEMI in HICs [29]; however, tailored recommendations are needed for LMICs to address unique challenges that make adoption of these guidelines difficult. Recently, a multi-country collaboration of investigators [29] created a consensus document to address the problems, gaps in knowledge, and suggest solutions for STEMI care in LMICs. We hope that contemporary data, such as those from the current study, and real-world studies will inform future investigations, guidelines, and development of systems of care to improve outcomes among patients with STEMI in LMICs.

## Study strengths and limitations

This study has a number of limitations. First, this is a retrospective study of a randomized trial and thus residual confounding cannot be excluded even after propensity matching. Also, of 13,689 STEMI patients, 3,837 did not have documented reperfusion. It unclear whether these patients did not undergo reperfusion or there was insufficient documentation, which can be a source of bias. Second, since fibrinolysis administration, door-to-needle, and needle-to-balloon times were not complete and not adjudicated, the nature of nPPCI was not well defined and included PhI, facilitated and rescue PCI. While this reflects contemporary practices in managing STEMI, it does not help to delineate the strength and weakness of each approach. This would have served as an important parameter for STEMI-care systems in Kerala, India adjudication of all conceivable endpoints was challenged by the large number of hospital (n = 63) and the resource-limited setting. Additionally, we were unable to obtain information on socio-economic resources (e.g. income, education, etc.), which may have provided additional information to guide propensity matching and insights into differences in reperfusion strategies and outcomes in this population. On the other hand, our study had a number of important strengths including a large STEMI patient sample from a LMIC with information on contemporary reperfusion strategies, multiple potential confounders, and details regarding cardiac presentation.

## Conclusion

In a large, contemporary population of patients from a LMIC, a nPPCI reperfusion strategy was associated with comparable short- and intermediate-term outcomes relative to a PPCI strategy. LMICs face numerous challenges establishing and sustaining STEMI-care networks, and incorporation of nPPCI reperfusion strategies may be a reasonable approach in these settings. Further studies are needed to understand practice variations and outcomes associated with contemporary, real-world reperfusion strategies in LMICs that can be used to inform guidelines for acute management of STEMI in resource-limited settings.
